# Natural antisense RNAs as mRNA regulatory elements in bacteria: a review on function and applications

**DOI:** 10.1186/s11658-016-0007-z

**Published:** 2016-07-28

**Authors:** Fatemeh Saberi, Mehdi Kamali, Ali Najafi, Alavieh Yazdanparast, Mehrdad Moosazadeh Moghaddam

**Affiliations:** 1 Molecular Biology Research Center, Baqiyatallah University of Medical Sciences, Tehran, Iran; 2 Nanobiotechnology Research Center, Baqiyatallah University of Medical Sciences, Tehran, Iran; 3 Applied Biotechnology Research Center, Baqiyatallah University of Medical Sciences, Tehran, Iran

**Keywords:** Bacteria, Natural antisense, *Cis-*asRNA, *Trans*-asRNA, Regulation, Riboswitch, Hfq protein, RNA engineering, RNA silencing, RNA thermosensors

## Abstract

Naturally occurring antisense RNAs are small, diffusible, untranslated transcripts that pair to target RNAs at specific regions of complementarity to control their biological function by regulating gene expression at the post-transcriptional level. This review focuses on known cases of antisense RNA control in prokaryotes and provides an overview of some natural RNA-based mechanisms that bacteria use to modulate gene expression, such as mRNA sensors, riboswitches and antisense RNAs. We also highlight recent advances in RNA-based technology. The review shows that studies on both natural and synthetic systems are reciprocally beneficial.

## Background

### Regulatory antisense RNA

Antisense RNAs (asRNAs), also referred to as natural regulatory RNAs, are small molecules that perform their regulatory function by recognizing sequence and structural elements that are present in themselves and their target mRNAs. AsRNA-mediated regulation generally inhibits mRNA transcription and/or translation or induces their rapid degradation. In fewer cases, asRNAs activate expression of mRNAs [1, 2].

We are using the terms regulatory asRNAs and natural regulatory RNAs to mean a diverse group of bacterial RNAs that modulate a broad range of physiological responses through complicated and delicate controlling mechanisms [3]. Although natural regulatory RNAs were first discovered in bacteria in 1967, their importance and prevalence have not been appreciated for till recently [4]. These natural regulatory RNA sequences or transcripts are similar to the eukaryotic microRNAs (miRNAs) in terms of function and properties. However, bacteria do not possess RNA interference machinery (RNAi) per se. Bacterial asRNAs and eukaryotic miRNAs both target mRNAs to regulate their translation and/or degradation [1, 5].

In recent years, the prevalence of long non-coding RNAs (lncRNA) and natural antisense transcripts (NATs) has been reported in a variety of organisms. In general, antisense transcript regulatory mechanisms affect different levels of gene expression including: transcription interference, transcription attenuation, translation stimulation or inhibition, and RNA stability [6]. Another major discovery found in bacteria was that many regulatory RNAs dwell in mRNAs [1].

Here are some of the characteristics of this type of RNA.

- Antisense RNA can be inherited during conjugation (horizontal gene transfer).
- High-throughput transcriptomics has shown that every organism can potentially possess one or several asRNA regulators for every single gene.
- New asRNAs evolve easily and automatically during mutations of the original DNA template.
- Because both antisense and target RNAs are transcribed in close proximity due to the position of the *cis* on their templates, a high local concentration of both types of molecule takes place. These so-called steric effects and the limited diffusion of transcripts lead to an efficient interaction between antisense and target RNAs, which is suggested to be controlled and biologically effective.
- The spatial closeness of the promoter sites to antisense and target RNAs also causes transcriptional interference, and thus has a regulatory role.

On the other hand, asRNAs can also be defined as endogenous RNA molecules containing complementary sequences to the original transcripts (mRNAs). These endogenous asRNAs have been observed widely in both prokaryotes and eukaryotes [7].

### *cis-* and *trans*-antisense RNAs

Studies have shown that some asRNAs regulate protein function, mimicking the nucleic acids that are the regulatory targets of the protein, so they involve the protein in an inactive complex, whereas the majority of asRNAs pair with target mRNAs and shift their stability and/or translation [8]. In bacteria, these base-pairing/non-coding regulatory RNAs are generally subdivided into three groups: (i) *cis*-acting 5’ element non-coding asRNAs; (ii) *trans*-acting small non-coding asRNAs; and (iii) *cis*-encoded asRNAs.

A *cis*-acting 5’ non-coding asRNA is usually attached to the 5’ side of an mRNA, the expression of which is regulated by the non-coding RNA. A structural change in the non-coding RNA occurs through binding to small metabolites (riboswitches) or through change of temperature (thermoregulators) or pH (pH sensors). The structural change influences the transcription or translation of the downstream gene or genes in an operon.

*Trans*-acting small non-coding asRNAs are usually encoded in intergenic regions on the chromosome and control translation or degradation of their target mRNAs. Generally, each *trans*-acting non-coding asRNA has multiple target mRNAs and binds near the ribosomal binding site of the target mRNAs.

**Fig. 1:**
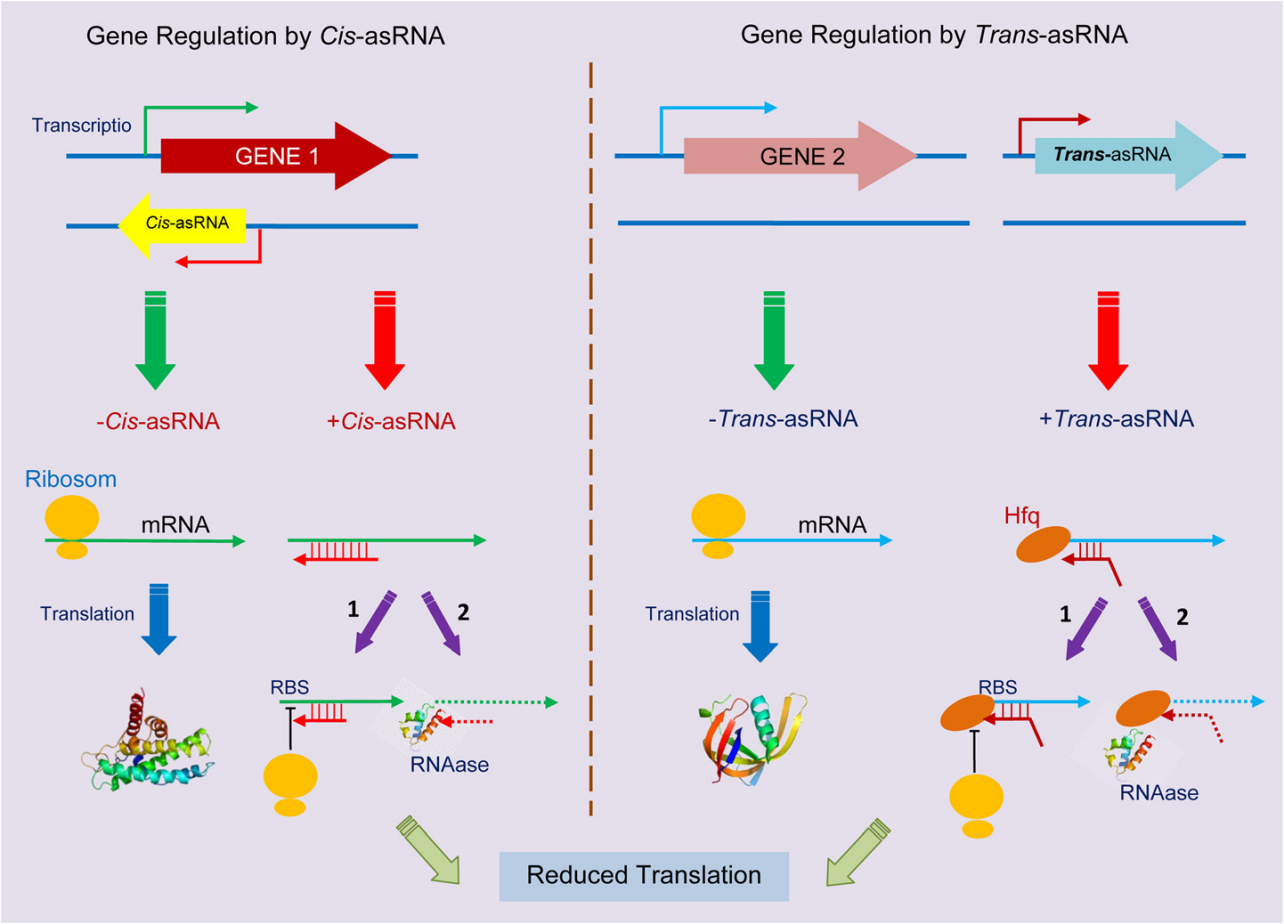
Gene silencing by natural antisense RNAs in bacteria. A – Cis-antisense RNAs (cis-asRNAs) are encoded with high degrees of complementarity to the target mRNA. B – Trans-antisense RNAs (trans-asRNAs) are encoded with limited complementarity to the target mRNA. In some species, trans-asRNAs require an RNA chaperone (Hfq) to facilitate binding to the target mRNA. Generally, in either case, once the asRNA is bound to the target mRNA, translation of the target gene is silenced by inhibition of the ribosome binding to the target mRNA (1); by induced RNase degradation of the asRNA–mRNA hybrid (2); or by a combination of the two processes

*Cis*-acting asRNA is expressed as a complementary sequence of mRNA that becomes the sole target RNA. Therefore, *cis-* and *trans*-acting non-coding asRNAs are a major part of the asRNAs in bacteria (Fig. 1).

Prokaryotes also have *cis*-encoded asRNAs. They significantly contribute to different biological procedures, such as genomic imprinting, circadian rhythm, cardiac gene regulation and recombination of antigen receptor genes [7, 9].

Some principal features of *cis*- and *trans*-asRNAs are summarized in Table 1. As implied therein, *cis*-asRNAs act on their targets through complete or near-complete complementarity, while short regions of complementarity suffice for *trans*-asRNAs to affect their target mRNAs. Therefore, *cis*-asRNAs form much more energetically stable duplexes with their targets than *trans*-asRNAs. Generally, *cis*-asRNAs are associated with mobile genetic elements such as plasmids, phages and transposons. They contribute significantly to the regulation of plasmid copy number, conjugation, phage life cycle and transposition. Several anti-toxicity activities have also been reported for some *cis*-asRNAs [1, 10].

**Table 1:** Differentiation of *cis*- and *trans*-asRNAs

*cis*-asRNAs	*trans*-asRNAs
Also known as *cis*-NATs or *cis* effector asRNAs	Also known as *trans*-NATs or *trans* effector asRNAs
Transcribed from the opposing DNA strand of the target gene at the same genomic locus	Transcribed from a separate locus to the target gene
Display perfect or extensive sequence complementarity with the target gene	Display imperfect or short stretches of complementarity with the target gene
Target individual mRNAs	Target multiple sense targets and form complex regulation networks
Mostly short	Frequently longer: several hundred nt, in some examples several kb long

*Cis*-asRNAs were first detected in viruses, then in prokaryotes and finally in eukaryotes. According to their relative orientation and degree of overlap, they are classified into 3 categories; head-to-head (5’ to 5’), tail-to-tail (3’ to 3’) and fully overlapping. Tail-to-tail orientation seems to be the most common type. Overlapping transcripts might comprise two protein-encoding genes, one protein-encoding and one non-encoding gene, or two non-encoding transcripts [11].

Based on recent studies, one of the popular *cis*-acting regulatory regions of the mRNAs acts as a potential target for the development of novel anti-bacterial compounds or as a regulatory factor for biotechnological applications due to versatile properties of the RNAs and their ability to be regulated by physical parameters (thermosensors) or small molecules (riboswitches). Other high-throughput screens have made it possible to design artificial metabolite analogs that modulate *trans*-acting regulatory RNAs with two functions: for example, a riboswitch coupled to an asRNA [12].

Thus, it is known that asRNAs regulate prokaryotic and eukaryotic gene expression at multiple levels including transcription, RNA editing, post-transcription and translation. They show such a wide distribution and conservation that the assumption of their accidental existence can be completely disclaimed (Table 2) [13–16].

**Table 2:** Some mechanisms used by bacterial antisense RNAs [16]

Antisense/target	Organism	Mechanism	Description	Needs Hfq
MicA/*ompA*	*Escherichia coli*	Direct (TIR) inhibition of translation	*Trans*-encoded; entails RNase E-dependent mRNA decay	Yes
SgrS/*ptsG*	*E. coli*	Direct (TIR) inhibition of translation	*Trans*-encoded; SgrS also encoded o protein	Yes
CopA/CopT	*E. coli* R1-type plasmids	Inhibition of translation	*Cis*-encoded; involves translational coupling	No
IstR1/*tisB*	*E. coli*	Inhibition of translation	*Trans*-encoded; binding upstream of TIR inhibits standby ribosomes	No
RNAIII/*sa1000*	*Staphylococcus aureus*	Inhibition of translational initiation and induced RNase III cleavage	*Trans*-encoded; full inhibition requires both translation block and endocleavage; also contain open reading frame	No
DsrA/*rpoS*	*E. coli*	Activation of RpoS translation	*Trans*-encoded; binding upstream of TIR	Yes
RNAIII/*repR*	*Streptococci* plasmid plP501	Riboswitch-like induction of premature transcription termination	*Cis*-encoded	?
GadY/*gadXY*	*E. coli*	Induced cleavage, stabilization of *gadX* mRNA	*Cis*-encoded between gadX and gadY	Yes
OOP/*cll*	*E. coli* Lambda phage	Induced RNase III-mediated cleavage near 3’ end	*Cis*-encoded; causes subsequent cll mRNA decay	?
MicC/*ompD*	*Salmonella*	Direct targeting for mRNA decay	CDS-internal target; no effect on translational initiation	Yes
RNAI/RNAII	*E. coli* ColE1-type plasmids	Inhibition of replication primer maturation	*Cis*-encoded; induces RNA folding change	No
crRNAs/phage RNA/DNA?	Bacteria and archea	Mechanism is homology-dependent but unknown	CrRNAs processed from precursor RNA; anti-phage immunity	?

*Trans*-asRNAs are generally much longer than the *cis*-asRNAs. It follows that the promoter and first transcribed nt of asRNAs may play the part of an intergenic spacer and then extend to a complementary region. However, *cis*-asRNAs form more stable complementary duplexes because of their binding kinetics. In addition, *cis*-asRNAs seem to exert their target interaction independently of proteins such as Hfq (Table 2), but other protein factors such as RNA helicases and RNA binding proteins are involved in the association between sense and antisense RNA [13].

The sizes of asRNAs are also very diverse. There are examples of rather short NATs of only 100 to 300 nt e.g., SymR, GadY and SyR7, but many asRNAs are substantially longer, ranging from 700 to 3,500 nt. At least one example, in *Prochlorococcus* sp. strain MED4, is 3500 and/or 7,000 nt, overlapping 14 genes of a ribosomal protein operon. These asRNAs protect a set of mRNAs that accumulate during phage infection from RNase E degradation. It is noteworthy that this type of asRNA–mRNA duplex formation masks single-stranded recognition sites of RNase E, leading to increased stability of the mRNAs during phage infection [17, 18].

In this review, we will focus our discussion on bacterial small RNAs that act as regulators. In general, the aim of this review is to provide an overview of some natural RNA-based mechanisms, such as mRNA sensors, riboswitches and asRNAs, which are used by bacteria to modulate gene expression. We would also like to highlight recent advances in RNA-based technology. This review emphasizes how studies on natural and synthetic systems are reciprocally beneficial.

#### Antisense RNAs in bacteria – discovery and history

The first natural *E. coli* asRNA (micF RNA) was identified during the characterization of the *E. coli* outer membrane porin ompC. During that study, it was noticed that several ompC promoter clones repressed the synthesis of the other major outer membrane porin OmpP. The responsible region for this activity turned out to be a 300-bp fragment located upstream of the ompC promoter, 70 % homologous to the DNA encoding the 5’ end of the ompF mRNA (including the ribosome binding site and the ompF initiation codon). Further studies showed that this homologous 300-bp fragment is transcribed in the opposite direction from ompC to a 174-nucleotide RNA that complements and encompasses a region of the ompF mRNA containing its translation initiation site. Based on this study, it was proposed that micRNA forms a hybrid with ompF mRNA and inhibits its expression by preparing a model that seeded the idea of using artificial micRNAs to regulate selected genes [19, 20].

Studies have shown that the *micF* gene produced this asRNA. *micF* is a stress response gene found in *E. coli* and related bacteria. It post-transcriptionally controls the expression of the outer membrane porin gene ompF by encoding a *trans*-asRNA (93 nt) that binds its target ompF mRNA and regulates ompF expression by inhibiting translation and inducing degradation of the message [21].

Several mapping, cloning, sequencing and bioinformatics techniques and tools have helped to identify and characterize asRNAs, but various aspects of their functions, structures and mechanisms of action remain to be explored. Accordingly, fundamental and mechanistic studies aiming to characterize novel regulatory RNA are needed if biotechnological and medical aims are to be met [22].

Although regulatory RNAs and their mechanisms were discovered in prokaryotes in 1967, it is only now that the central role of RNA in prokaryote gene regulation in all three domains of life can be demonstrated. The research that led to this discovery occurred in three major phases: 1967 to early 2001; 2001 to recent times; and current research [13, 23].

In 1967, Hindley identified a distinct and abundant RNA species, later named 6S RNAI. Its function in RNA polymerase activity regulation had been determined previously. The first *trans*-acting regulatory RNAs were discovered around this time [4].

Initially, it was thought that *cis*-asRNAs controlled the life cycle or copy number of extra-chromosomal genetic elements, bacteriophages, transposons and plasmids. One of the first identified antisense transcripts belonged to the gene cro in bacteriophage λ. It was revealed to be transcribed by bacteriophage λ when overexpression of the 77 nt OOP antisense transcript resulted in its co-degradation with the cII mRNA [24]. Another extra-chromosomal element was a *cis*-asRNA I involved in the regulation of maturation of the ColE1 primer and the control of plasmid incompatibility of ColE1-type plasmids.

Over the next two decades, although a small number of chromosomally encoded small RNA regulators such as MicF, DicF and OxyS were discovered, their fundamental importance and privileges were not adequately considered. Only 12 small RNAs containing entities like 6S RNA, tmRNA, RNase P RNA and 4.5 S RNA had been identified in *E. coli* by early 2001.

In 2001, considerable progress took place thanks to the application computational techniques and predictive approaches to complex systematic experimental screens. For example, comparative genome analysis of closely related species was used for searching transcriptional signals within intergenic regions and scoring the conservation of predicted RNA secondary structure with the aim of small RNA prediction in Enterobacteria. During this second phase, newly identified *trans*-acting asRNAs supplied abundant data for later functional characterization studies [25].

The advent of RNA-seq technology and its use in prokaryotic RNA studies triggered a new wave of discovery. This has led to new standards in the accelerated identification of transcripts and transcriptional start sites [13, 26].

#### Why antisense regulation?

Ongoing studies present two reasons for the advantage of asRNA regulation over other methods of regulation. First, asRNAs could provide an advantageous control system when a particular protein such as transposase needs to be expressed under very selective circumstances or repressed at a very tight level. Second, asRNAs provide one more level of control in the extensive regulation of their targets. For example, translation and expression of MgtC, a virulence factor of *Salmonella enterica*, is regulated at the transcriptional level by PhoPQ, and at the level of protein stability by the MgtR peptide. Additionally, it might be regulated at the post-transcriptional level by the AmgR asRNA [27].

In this way, the advantages of RNA-based regulation over classical protein-mediated transcriptional control can be referred as:

1. The response to the regulator happens in a shorter time because in this way mRNA is a direct target.
2. In the case of small RNAs, a low number of base pairs provide specific and fast mRNA recognition and binding.
3. Rapid specificity can be attained because a single nucleotide change suffices for a specificity shift.
4. The capacity of RNA regulators to modulate their conformation upon binding increases the number of contacts with a given ligand or makes the recognition of multiple targets easier [26].

These features show the potential of artificial mRNA regulators that can be beneficial for various biotechnological applications, supporting the need for more studies [24].

### Antisense RNA functions in bacteria

As mentioned, asRNAs affect cellular functions through transcription attenuation, translation inhibition, and regulation of plasmid copy number through the inhibition of primer maturation, prevention of formation of an activator RNA, and promotion or inhibition of mRNA degradation [16, 28]. They perform their regulatory actions by annealing to complementary mRNA strands. For example, they can block translation through steric hindrance or cause rapid degradation by dsRNA-specific RNases. The length and structure, intracellular concentration of the antisense sRNA and resistance to the degradation of the target mRNA influences the proficiency of degradation [13, 29]. A few examples of different asRNA mechanisms of action in prokaryotes are given below.

#### Induction of a bacterial gene

*MucD* gene expression and consequent alginate (biofilm) biosynthesis in *Pseudomonas aeruginosa* is regulated by *cis*-asRNA (named mucD_AS). Functionally, mucD_AS turned out to be able to induce biofilm formation in *P. aeruginosa*. Considering the biotechnological and medical significance of alginate as a key virulence factor of *P. aeruginosa*, this finding is particularly important [30].

#### Inhibition of bacterial gene expression

An asRNA named *micF* inhibits the expression of the membrane porin gene, *ompF*. This occurs under the regulation of environmental and internal stress factors, including elevated temperature, exposure to salicylate and redox stress. *ompF* is at its maximal expression level at low temperature and osmolarity and this is regulated at the transcription and post-transcription levels by *micF* asRNA [16]. However, the regulatory mechanism is not clear: it seems that micF destabilizes the *ompF* mRNA and inhibits its translation. It is likely that specific binding proteins are also associated to micF asRNA, while both seem conserved in other Gram-negative species. Other controlling systems are involved at different levels [31, 32].

#### Post-transcriptional inhibition of mRNA

dicF is an asRNA that inhibits cell division in *E. coli*. ftsZ mRNA, which is apparently involved in initiating division, is inhibited by several factors, including the 53-nt dicF-RNA, which is transcribed from *dicB* operon (another inhibition factor) and partially complementary to the ftsZ mRNA [31].

#### Inhibition of translation

The expression of glutamine synthetase gene (*gInA*) is likely regulated by asRNA in *Clostridium*. This is an important enzyme for nitrogen assimilation in Gram-positive *Clostridium acetobutylicum* and is nitrogen regulated. The regulatory 43 nt asRNA is complementary to the *ginA* RBS and its transcription induced in nitrogen level of environment is high leading to repressed *ginA* transcripts [31, 33].

#### Regulating plasmid replication

Controlling genes and sites in the replication frequency of plasmids are located on the plasmids themselves, which keeps the copy numbers of plasmids stable. Many plasmids use asRNAs to measure their own copy numbers and adjust deviations. Controlling other protein synthesis (such as a rate-limiting replication protein) or primer inhibition (in the case of the ColE1-type plasmids) can take place too [19, 31, 34]. Some examples are given in Table 3.

**Table 3:** Plasmids using NAT regulating systems

Plasmid	The role of the antisense RNA
ColE1	An asRNA, termed RNA I, binds to the 5’ end of RNA II, triggering a conformational change that initiates inhibiting replication by preventing persistent hybrid formation.
	Another asRNA, Rcd^a^, complementary to the Cer region of plasmid, is synthesized when synaptic complexes between Cer sites are formed. It inhibits division when the plasmid is in a multimeric state and at risk of being lost.
IncFII-Like	An asRNA blocks the translation of leader protein and causes inhibition of Rep protein synthesis.
IncIu-IncB	An asRNA prevents the formation of an activator RNA pseudo knot.
pT181 and pIP501	Antisense RNA induces attenuation of transcription.

^a^Repressor of cell division

#### Inhibition of F-like plasmid conjugation

More than 30 *tra* genes are involved in the conjugation of F-like plasmids (such as *Incf* and *R1*). One of these is a transcriptional activator, traJ. It is regulated by an asRNA system, FinP, which complements to *traJ* RBS and blocks its translation. FinP metabolic stability increases with the help of a ribonucleolytic degradation inhibitor protein, FinO. Structurally, FinP has two stem-loops, so loops are responsible for specificity and stems and play a pivotal role in its activity [4, 14].

#### Inhibition of plasmid and host-encoded killer systems

Host-killing systems are employed by many bacterial plasmids to kill cells that fail to keep at least one plasmid copy at cell division to guarantee their maintenance in bacterial cells [19, 31]. All members of the growing list of plasmid-borne killer systems have a similar genetic structure. They produce a small protein that increases the permeability of the cell membrane, leading to cell death. There is an asRNA that inhibits the expression of this protein at the post-translational level. An overlapping reading frame is involved in the killer mechanism and in its inhibition [35].

#### Control of bacteriophage development

In the bacteriophages P22 and A, antisense control exists as a secondary control strategy and contributes at various levels to their developmental pathways. Some of these RNA transcripts and their functions are listed below [31].

- OOP RNA facilitates cell mRNA decay.
- PaQ RNA inhibits late gene expression.
- P22 sar RNA inhibits anti-repression.
- Sas RNA in phage P22 regulates alternative translation.

#### Controlling toxin synthesis

SymE protein shows homology to MazE, an antitoxin, encoded from an SOS-induced gene. It is suggested to be involved in cell growth inhibition, decreased protein synthesis and increased RNA degradation, and therefore functionally resembles RNA endonuclease toxins. SymR is one of several mechanisms that repress SymE gene expression. It contains an asRNA that is encoded in *cis* to the *SymE* gene [36, 37].

#### Small RNAs involved in quorum-sensing and biofilm formation

Recent studies have revealed that RNAs are key regulators in pathogens. Small RNAs regulate the translation and/or stability of mRNAs that encode virulence factors, or proteins with roles in adaptive responses, which are triggered by environmental cues and stresses. Generally, the transcription of many pathogenesis-related RNAs is dependent on the growth phase but in several pathogens, the secretion of virulence factors is regulated by cell-density sensing (quorum sensing), a process that involves communication through secreted signaling molecules. Several regulatory RNAs are the main effectors of quorum-sensing systems [38, 39].

For example, in *Vibrio cholerae*, the sensory signals converge on a response regulatory protein, LuxO. At low cell density, when the quorum-sensing autoinducer is absent, phosphorylated LuxO activates the transcription of four redundant Qrr RNAs as quorum regulatory RNAs (Qrr1–Qrr4) that regulate the mRNA of the downstream target gene *hapR* through the inhibition of translation and mRNA degradation. In *Staphylococcus aureus*, the effector of quorum sensing is encoded by the *agr* system, which is composed of two divergent transcription units. The first operon combines a density-sensing cassette including *agrD* and *agrB*, and a two-component sensory transduction system (agrA and agrC), which is required for its autocatalytic activation as well as for the activation of transcription of RNAIII, the intracellular effector of the *agr* regulon.

Although it had previously been thought that small RNAs are noncoding, based on this research several such entities were recognized to be both regulatory and protein coding (regulatory RNA and mRNA). For example, RNAIII is one of the first identified *trans*-acting regulatory RNAs shown to encode a 26-amino acid peptide that may be involved in biofilm integrity in addition to its regulatory effect in quorum-sensing in *S. aureus* [40].

In *E. coli*, when phosphor–sugar intermediates can increase to toxic levels, SgrS, a 227-nt small RNA, represses *ptsG*, the glucose transporter component of the PTS, at the translational level. SgrS encodes for a 43-amino acid oligo-peptide, SgrT, which inhibits glucose transportation of PtsG. Transcriptional profiling has revealed five potential sRNAs in *Listeria monocytogenes*. They are suggested to encode 28-to 64-amino acid small peptides [41].

### A brief overview of other regulatory RNA elements

Many other kinds of mRNA regulatory elements exist that are actually located on the same strand and near the locus of target mRNA, just upstream of the target coding sequence. These types of regulatory elements are also classified as *cis*-acting RNA regulators [42].

#### Metabolite-sensing riboswitches

Some regulatory mRNAs modulate gene expression through allosteric mechanisms due to stimulant metabolites that modify the mRNA structure and activity by binding to 5’ UTR regulatory elements. The modified structure of the mRNA influences the expression of the following coding sequence. These metabolite-sensing mRNAs, which are called riboswitches, are widespread in bacteria. They give feedback regulation that supplies the cell with a reciprocal regulation system that is responsive to the concentration of stimulant metabolites. In addition, the synergism of different riboswitches in functional units develops even more complex regulatory arrangements. Such metabolite-specific tandem riboswitches may act independently or associate together [1, 12].

#### RNA thermosensors

RNA molecules have a particular potential to sense temperature due to their versatility and dynamism. Several mRNAs of heat- and cold-shock proteins also act as thermosensors. It is shown that some proteins associated with bacterial pathogenesis or bacteriophage development and lysogeny have mRNAs that are regulated by the thermosensor system. As with the riboswitches, the thermosensor regulatory elements, are settled at the 5’ UTR of mRNAs. Another mutual mechanism controls all heat- and cold-shock responsive elements and translation initiation, so that low temperature causes target mRNA RBS to fold into a helical structure preventing the formation of the ribosomal initiation complex and protein synthesis initiation. However, in higher temperature, the inhibitory structure unfolds and then protein synthesis begins after ribosomal initiation complex formation.

In many Gram-negative bacteria, this 5’ UTR regulatory element of heat-shock protein mRNAs contains 4 hairpins. It has the name ROSE (repression of heat-shock gene expression). Another example is the 5’ UTR of prfA mRNA in *Listeria monocytogenes*, which contains a similar hairpin that melts at host temperature (37 °C), permitting the protein to start its virulence function. Some other thermosensitive elements may adopt more stable structures. Thus, thermosensitive regulatory activity is influenced by the rate of the transcription, the thermosensitive folding performance and the ribosome recognition efficiency of these structures [1].

#### Hfq-associated regulatory small RNAs

As implied before, thermosensors and riboswitches were from well-characterized groups of *cis*-acting regulatory sRNAs. *Trans*-acting regulatory RNAs are the best-characterized class of RNA regulators [43, 44]. *Trans*-encoded asRNAs are extensively observed in bacterial genomes and show partial complementarity to their target. The pleiotropic regulator protein Hfq is a highly conserved hexameric protein that tends to bind to an AU-rich region of a single-strand RNA preferentially close to a stem-loop structure. It was recently found in association with many *trans*-encoded regulatory RNAs, regulating the mRNA translation [45, 46]. Most of the Hfq-dependent small RNAs contain 3 structural partitions: a 5’-targeting domain, a Hfq-binding domain and a 3’-located terminator [28].

### Synthetic antisense RNA

An antisense RNA regulatory system is potentially very useful for metabolic engineering and/or as a therapeutic agent in prokaryotic organisms [27]. The understanding of regulatory asRNAs and their mechanisms enable us to design synthetic RNA regulatory systems for a variety of biotechnological and medical purposes. Artificial regulatory RNAs have aided many biological studies, such as in the identification of riboswitches in bacteria.

In some studies, the targeting region of recognized Hfq-associated regulatory asRNAs was merged to other asRNA backbones to construct custom regulatory asRNAs with a desired regulatory function. As the library of various regulatory small RNA modules becomes more complete, the design of a completely recombinant asRNA becomes more approachable. Mutational techniques and in silico modeling software are helpful to predict the designed asRNAs structurally and functionally [1, 47]. What follows are some of the existing applications.

#### Antisense RNA as a metabolic engineering tool to enhance the productivity of several bacterial hosts

Natural prokaryotic antisense systems were the first RNA-based regulatory systems applied in bacteria. The locus of *parB* partition in the plasmid R1, which stabilizes the plasmid in the carrier cell through toxin/antitoxin counteraction, is also regulated under an RNA-based regulation system. Therefore, this regulatory system might be helpful when R1 is used to produce a special product [48]. In addition, there are many other biological procedures that are controlled with asRNA-based strategies. The regulation of biosynthesis of the global regulatory proteins and metabolites in *Clostridium acetobutylicum* and *E. coli* are noteworthy examples [14].

Furthermore, asRNA strategies are suggested to enhance recombinant protein production in *E. coli*. One approach is to silence the RNaseE using asRNAs and decrease the cellular level of RNaseE. This is supposed to decrease mRNA degradation in the cell, resulting in augmentation of the target mRNA and the related product [49].

Regulatory asRNA was also studied in *Clostridium acetobutylicum*. The idea was to redirect the primary metabolism of bacteria trying to reduce the levels of the butyrate-forming enzymes, because butyrate is assumed to induce solvent genesis in *C. acetobutylicum*. The larger goal was downregulation of the primary metabolism of *C. acetobutylicum*, which is a promising organism for biorefinery [50, 51].

Regulatory asRNA was also used in *Lactobacillus rhamnosus*, a milk-fermenting bacterium. The purpose was to change the polysaccharide size without any alteration to the total amount. This offers a conditional control of gene expression in metabolic engineering samples without gene inactivation [52].

#### Antisense RNA as bacterial protection against bacteriophages

Bacteriophages can be highly disruptive in microbial industries that use starter cultures or produce live mucosal vaccines or enzymes and metabolites. It has been shown that asRNA anneals to sense RNA and makes it subject to dsRNase enzymes. The idea of designing asRNAs against essential genes for phage development is of great interest [53].

#### Artificial antisense RNAs as gene silencers

Despite all of the controversial reports on the ability of asRNAs to silence genes, one study did show that they can silence a gene by decreasing target mRNA concentration [54]. Since lacZ is an ideal subject for gene silencing studies, it has been used as a target for synthetic asRNAs. The β-galactosidase activity assessment has shown to be successful in silencing lacZ in *E. coli*. The goal of the study in question was to survey the silencing efficiency and repression mechanism [54, 55].

#### Synthetic RNA silencing as antimicrobials

A variety of RNA-level regulatory systems have been developed in bacteria. The most important factor in their development is that mRNA can rapidly switch from repression to expression and vice versa in response to environmental stimulation [56, 57]. The antibiotic resistance system is noteworthy because cell survival in the presence of antibiotics needs a rapid antibiotic resistance gene expression and *cis*-encoded antisense sequences provide this. These asRNA coding sequences are situated within a short open reading frame just upstream of the start codon region of both the chloramphenicol and erythromycin resistance genes [58, 59]. RNA-silencing sequences manage gene regulation not only located out of the operon but also when they lie within the operon. For instance, Spot42 RNA, an antisense repressor sequence for gal is located within the galETKM operon. Additionally, RNA silencing acts as an RNA regulatory system in the protection of bacteria from viral infection. Natural RNA silencing provides bacteria to a wide range of RNA-level gene control [60].

#### Antisense RNA as a tool to analyze essential genes in bacteria

Antisense RNA regulation has been employed for determining essential genes of bacteria. *S. aureus* has been targeted by two research groups applying shotgun technology to make a library of *S. aureus* genome fragments cloned under inducible promoters aimed to essential gene identification. Although the suboptimal regulatory efficacy of RNA may contravene the desired lethal activity, the RNA regulators have been successful enough to recognize many essential genes of *S. aureus*, leading to infection resolution in the animal model [1, 61].

#### ncRNAs/RNA aptamer-fused regulators

Non-coding RNAs (ncRNAs) have a variety of regulatory functions in cell. Since most *trans*-acting ncRNAs generally cannot sense cellular signals directly, allosteric RNA fusion molecules have been engineered combining ncRNAs (as the target recognition motif) and RNA aptamers (as the ligand-sensing motif). The fused aptamer part enables the ncRNA modulatory activity in *E. coli*. This is applicable to both translation ncRNA regulators (e.g., IS10 ncRNA) and transcription ncRNA regulators (e.g., pT181 ncRNA). This reconfiguration develops an orthogonally acting fusion molecule capable of recognition of different ligands and affecting different subjects in a biological system [62–64].

Finally, the technique of aptamer–ncRNA fusion molecules provides researches with a very extended and efficient tool for ligand-sensing regulatory circuits. A single ncRNA can be fused to various aptamers as sensing domains and can bring up ligand sensor control systems that respond to multiple input signals applicable for structurally related families of ncRNAs [65].

#### Synthetic small RNAs as ligand-specific purification tools

For experimental investigations of small RNAs and their interactions with proteins, they must be purified in a native form. Aptamer-tagged variants of target small RNAs can be produced and used in RNA-based affinity chromatography. Thus, appropriate plasmids have been developed to express target small RNAs tagged with one of three global aptamer sequences: MS2, boxB or eIF4A [65].

#### Synthetic RNA thermosensors

By exploiting biomimicry, in vivo selection and computational design, synthetic thermosensors have been created. These trials made a simple on/off switch to express any desired gene in response to temperature. A similar principle was implemented to fuse the PrfA leader sequence to *gfp* mRNA. It could confer temperature dependence to gene expression in *E. coli* [66].

Other physical stimuli can also potentially be detected by this sensor-mediated regulatory system, making it possible to apply more criteria to biological systems and offering more metabolic engineering tools to researchers [1].

RNA thermometers (RNATs) mostly regulate translation using a zipper like function and blocking the SD sequence at low temperature, resulting in suspension of translation initiation. As temperature increases, melting of the secondary structure causes the release of an SD sequence resulting in the resumption of translation [67].

A novel functionality was created based on a temperature-controlled element of *Salmonella* fused to a hammer-head ribozyme (HHR) that fractions itself to a liberated RBS and a thermo-sensitive hairpin connected to the HHR. It brought a temperature-controlled ribozyme forward – a so-called thermozyme. Therefore, at higher temperature, the self-cleavage activity of the thermometer structure is impaired and the gene expression is silenced. This is the opposite of the natural behavior of thermosensors [68].

Another simple theoretical thermometric mechanism was implemented for de novo design of a synthetic thermosensor based on an RBS embedded in a single stem loop that unmasks when the temperature reaches a predicted range. This hypothesis was assessed by constructing a particular modular structure including a promoter, a start codon, an SD sequence and a complementary anti-SD sequence (separated by four unique restriction sites), and standard reporter genes.

Another example is that the synthetic thermometer is designed based on the 5’ UTR of the *cIII* gene of coli-phageλ, which switches between two secondary conformations (on/off structures) in response to temperature rather than fully melting. In the off conformation at low temperature, the RBS is veiled, but in the on conformation at higher temperatures, the RBS is revealed. Designed RNA thermometers subjected to extra mutations in practice lead to functional thermosensors that were actually temperature inducible using the reporter gene of lacZ (encoding β-galactosidase) [66, 69, 70].

### Challenges and difficulties in using antisense RNA

Although RNA regulatory systems have many applications, some obstacles remain.

#### Method-born limitations in the procedure of small RNA identification

Currently, classical procedures of small RNA identification rely on bioinformatical approaches, which are somehow limited to a predefined data pool. This may direct research to an incomplete library that displays bias to sequence conservation, specific promoters and Rho-independent terminators, while missing several other aspects, including ORFs, Rho-dependent terminators and Hfq-independent small RNAs. To ensure a global unbiased approach to small RNA identification, other experimental and predictive procedures such as transcriptional profiling with the whole genome, massively parallel sequencing, and targeted identification of small RNAs are suggested [71].

#### Sufficient supplement of synthetic RNA for silencing

Another difficulty of the practical application of RNA silencers, either naturally existing in the cell or delivered into the cell, is the sufficiency of the silencer agent for efficient silencing activity. Considering the abundance of mRNAs in the cell, an acceptable level of expression or delivery and stabilizing structures should be provided in order to achieve practical silencing [60].

Most delivery methods are culture dependent. Most prokaryotes from natural environments are not readily culturable. Moreover, even in culturable in vitro genetic manipulation methods, the nucleobase polymer transferring process is not 100 % efficient. On the other hand, DNA and RNA transferred to natural microbial communities can change their functionality. However, in addition to their beneficial applications, the inevitable subsequences should also be considered [72].

#### Delivery methods

In the world of molecular biology, the success of every technique such as gene therapy and RNA regulation is highly dependent on the efficiency of the delivery approach of the multi-nucleotide molecule to the cell. A simple and efficient delivery system is necessary to exploit a prosperous RNA regulatory technology. Different biological physical and/or chemical methods are exerted for nucleotide polymers, as summarized in Table 4 [73–76].

**Table 4:** Delivery methods for transferring synthetic regulatory RNAs to bacteria

Delivery methods	Traits
Biological methods	
Transduction	A specific DNA donor is required for DNA transfer to recipient bacteria
Conjugation	Requiring physical contact of recipient and donor (host strain) with a conjugative plasmid or participation of a third bacterium with a helper plasmid
	Not useful for large-scale delivery applications
Gene transformation	Limited to a few naturally competent groups
Physical methods	
Electroporation	Highly efficient but requires low ionic strength medium and high voltage
	Not useful for large-scale delivery applications
Laser irradiation	Employs a laser to change cell permeability to allow transferal; requires physical contact of laser and cells
Ultrasound DNA delivery (UDD); Sonoporation	Appropriate approach for plasmid or DNA fragment transferal to eukaryotic cells (e.g., gene therapy)
Heat shock transfer	Mostly used for *E. coli* (in parallel with the calcium phosphate method)
Chemical methods	
Protein & Peptides	Introduced in the late 1950s, this technique originally used high salt concentration and polycationic proteins to enhance nucleic acid entry into the cell.
	Now cationic peptides are using to enhance nucleic acid delivery. Cationic peptides have been found useful for enhancing cellular uptake and/or cell targeting oligonucleotide analogs. These peptides are synthetically conjugated, used as non-covalent complexes, or used in combination with polymer formulation techniques
Calcium phosphate	Simple, effective and still widely used for nucleic acid delivery
Artificial lipids	DNA has been successfully complexed with cationic, anionic and neutral liposomes. These complexes can be handled easily, but lipid-based systems generally have significant drawbacks, including the lack of targeting and variations arising during fabrication
Naonparticles	Using carbon nanotubes, nucleic acid is delivered into cells. Magneto-transformation has also been used for nucleic acid transfer, but in that method, pulsed magnetic fields assisted the delivery of DNA using magnetic nanoparticles.

The most commonly used methods include conjugation, electroporation and heat shock transfer (especially for *E. coli*). Other physical techniques are usually only employed for small-scale prokaryotic cells [77]. Ultrasound DNA delivery (UDD) is another practical method that can theoretically deliver DNA or RNA to any cell type (including bacterial, fungal, plant or mammalian cells) without the need for ion-free media and are thus usable for growing cells in natural media or human body fluids. Finally, it provides a non-invasive method allowing no necessity of direct physical contact [78]. However, the frequency ranges that are efficient for eukaryotic cells are less effective in prokaryotic cells compared to classic bacterial transformation methods [73].

## Conclusion

The antisense RNA regulatory system is a mechanism for sequence-specific recognition of a particular transcript. It is usually involved in the degradation of said transcript. However, by definition, it could also be involved other forms of reduction or alteration of expression. New asRNA gene-controlling cases are being identified in nature, accompanied by the discovery of more mechanisms with a variety of potential applications. These utilities derive from variable mechanisms through which RNA regulatory systems influence target mRNA. AsRNAs can cause target mRNA degradation, change mRNA processing and/or affect mRNA transcription. The same applies to artificial asRNAs.

It remains to be seen whether transcriptional interference or antisense regulation can regulate sense–antisense pairs, but synthetic biology studies indicate that antisense transcription can improve existing networks by adding some constructive complexities. It is also possible to create new networks with desirable properties.

Regulatory RNA systems have considerable potential and are already used in silencing bacterial genes, altering bacterial behavior and inhibiting bacterial biofilm formation. They are also used as novel antibiotics, especially against multidrug-resistant bacteria. These applications have made them a popular subject for research.

## Abbreviations

asRNA, antisense RNA; dsRNA, double-strand RNA; HHR, hammerhead ribozyme; lncRNA, long non-coding RNA; miRNAs, microRNA; NAT, natural antisense transcript; ncRNA, non-coding RNA; RBS, ribosome-binding site; RNAi, RNA interference; ROSE, repression of heat shock gene expression-like thermometers; UDD, ultrasound DNA delivery; UTR, untranslated region
